# Nursing care of an elderly patient with toxic epidermal necrolysis: a case report

**DOI:** 10.3389/fmed.2026.1824922

**Published:** 2026-04-23

**Authors:** Danqiong Wang, Mengjiao Yuan, Ying Ying

**Affiliations:** 1 Department of Intensive Care Unit, Ningbo No. 6 Hospital, Ningbo, Zhejiang, China; 2 Department of Nursing, Ningbo No. 6 Hospital, Ningbo, Zhejiang, China

**Keywords:** critical care nursing, elderly patients, mucosal nursing, stage-specific nursing, toxic epidermal necrolysis, venous access management, wound care

## Abstract

Toxic epidermal necrolysis (TEN) is a rare, life-threatening mucocutaneous disorder, and its occurrence in elderly patients with compromised immunity and multiple comorbidities poses significant challenges to nursing care and prognosis. This paper summarizes the ICU nursing experience of an 84-year-old female patient with TEN presumably induced by parecoxib injection. The patient presented with 55% body surface area (BSA) skin involvement, combined with bedridden status due to fracture and electrolyte disturbances, with an expected mortality rate of 30–50%. An individualized nursing plan was formulated through multidisciplinary collaboration. Clinically, skin wound care was the cornerstone of management, incorporating refined, stage-specific interventions during the blister exudation, crusting, and healing phases, with yellow light irradiation and epidermal growth factor applied throughout all phases to promote wound healing. Targeted nursing measures were applied to vulnerable mucous membranes, particularly the oral cavity, eyes, and perianal region, including occlusive therapy and fecal diversion using ostomy bags to reduce local irritation. A midline catheter was prioritized, and an innovative adhesive-free dressing fixation strategy was adopted to ensure the safety of intravenous access. Full-process protective isolation and strict aseptic techniques were implemented to strengthen infection prevention and control. Meanwhile, medication nursing for immunomodulation, anti-inflammatory therapy, and symptomatic supportive care was delivered in accordance with the S3 Guideline: Diagnosis and Treatment of Epidermal Necrolysis (Stevens–Johnson Syndrome and Toxic Epidermal Necrolysis) developed by the German Dermatological Society (Deutsche Dermatologische Gesellschaft, DDG). Following 17 days of systematic nursing care, the patient’s wounds achieved nearly complete epithelialization, with no new blisters or exudation observed. Infection markers were significantly reduced, and the patient was successfully transferred to a rehabilitation ward. Her recovery duration was shorter than the average for similar patients, and no severe ocular complications developed. This case demonstrates that a comprehensive nursing strategy incorporating staged wound care, multidimensional mucosal protection, individualized intravenous access management, and full-process infection control can effectively reduce complication risks, accelerate wound healing, and improve outcomes in elderly patients with severe TEN, providing a reference for clinical nursing practice in this vulnerable population.

## 1 Introduction

TEN is a rare, acute, life-threatening mucocutaneous disorder predominantly induced by medications (1). Antibiotics and antipyretic analgesics are the most common causative agents. Its pathological hallmark is extensive apoptosis and necrosis of epidermal keratinocytes, accompanied by severe systemic symptoms (2). The main clinical manifestations include hemorrhagic erosions and crusts at mucocutaneous junctions such as the ocular, labial, and vulvar regions, or generalized erythema with epidermal necrosis (3). The disease is classified according to the extent of skin detachment: Stevens–Johnson syndrome (SJS) is defined as <10% of BSA involvement; SJS/TEN overlap involves 10–30% BSA; and TEN involves >30% BSA (4). The incidence of SJS is 3.96–5.03 per million, SJS/TEN overlap approximately 1–5 per million, and TEN 0.94–1.45 per million (5). TEN is prone to be complicated by multiple organ failure and carries a high mortality rate, with mortality rates of 5.4% for SJS, 14.4% for SJS/TEN overlap, and 15.3% for TEN (6).

In December 2025, our department admitted an 84-year-old patient with TEN. Following 17 days of treatment and nursing care in the intensive care unit (ICU), the patient’s wounds were nearly healed, with no new blisters or exudation, a significant reduction in infection markers, and successful transfer to a rehabilitation ward. In this case, skin care and infection prevention and control served as the core interventions. Staged wound care was implemented across the blister-exudative phase, crusting phase, and healing phase. Throughout the course of care, yellow light irradiation combined with epidermal growth factor was administered to promote wound healing. Targeted care was provided for mucosal and special anatomical sites, with occlusive therapy and colostomy bags for fecal collection to minimize local irritation. A midline catheter was selected as the preferred vascular access, accompanied by an innovative adhesive-free dressing fixation strategy. Medication administration was performed strictly in accordance with relevant guidelines, and full-process protective isolation was rigorously implemented. This was a rare case of severe TEN in an 84-year-old patient. The patient had a SCORTEN score of 3, combined with additional risk factors including advanced age, hypertension, and fracture. After 17 days of ICU management, the wound healing rate reached 95%, and the recovery period was shorter than the average for similar patients (7). No severe ocular complications occurred, verifying the scientific validity and effectiveness of the nursing strategy. In this study, an individualized nursing plan was formulated through multidisciplinary collaboration. The nursing experience is summarized as follows.

## 2 Patient condition

### 2.1 Basic condition

An 84-year-old female patient was admitted on November 28, 2025, due to “motor dysfunction of the left upper extremity and right lower extremity for 28 days after a traffic accident” and received limb function rehabilitation in the rehabilitation ward. Chief complaint: sudden generalized cutaneous erythema, blisters, and erosion, accompanied by fever during rehabilitation. History of present illness: On December 5, 2025, round erythematous lesions 1–2 cm in diameter appeared on the abdomen, back, and lower extremities. On December 8, large confluent blisters approximately 5 cm in diameter and dark erythematous patches coalesced over the trunk and extremities. On December 9, extensive epidermal detachment with marked exudation developed, with a maximum body temperature of 39.1 °C, accompanied by labial mucosal erosion and generalized pain. Past medical history: hypertension; no other chronic medical conditions documented. The patient had been bedridden for 28 days due to fracture and motor dysfunction of the left upper extremity and right lower extremity following a traffic accident. Family history: no familial genetic diseases. Psychosocial history: normal cognitive function and intact orientation; stable mood; cooperative behavior and good medical compliance; harmonious family relationships; family members provided emotional support and practical care, with a favorable family environment for rehabilitation. The patient received limb function rehabilitation in the ward after admission, without significant rapid improvement in motor dysfunction. During rehabilitation, she received continuous analgesic treatment with parecoxib injection for 23 days. Skin reactions were not monitored promptly during medication, and subsequent allergic-like reactions including cutaneous erythema and blisters occurred, which responded poorly to conventional anti-allergic therapy. The patient was then transferred to the ICU for intensive monitoring and treatment. Vital signs and laboratory findings on admission to and transfer from the department are detailed in Table 1.

**Table 1:** Vital signs and laboratory findings: ICU admission vs. transfer.

Vital signs/laboratory findings	Admission	Transfer
Body temperature (T)	38.4 °C	36.7 °C
Pulse (P)	98 bpm	88 bpm
Respiratory rate (R)	20 bpm	20 bpm
Blood pressure (BP)	149/69 mmHg	142/71 mmHg
Bicarbonate (HCO₃^−^)	20.2 mmol/L	21.2 mmol/L
Hemoglobin (Hb)	98 g/L	115 g/L
D-dimer	7080 μg/L	154 μg/L
Interleukin-6 (IL-6)	112.5 pg/L	17.4 pg/L
High-sensitivity C-reactive protein (hs-CRP)	142.8 mg/L	30.6 mg/L
Procalcitonin (PCT)	0.41 ng/L	0.11 ng/L
Albumin (ALB)	28.9 g/L	42.6 g/L
Serum potassium (K^+^)	2.9 mmol/L	4.2 mmol/L
Blood urea nitrogen (BUN)	15.3 mmol/L	7.28 mmol/L

### 2.2 Diagnostic assessment

The diagnosis in this case was established based on the patient’s medication history, typical mucocutaneous manifestations, SCORTEN score, and laboratory inflammatory markers, and was finalized through multidisciplinary consultation involving the Department of Critical Care Medicine, Department of Orthopedics, Department of Dermatology, and Department of Infectious Diseases. The early clinical manifestations of TEN are like those of allergic dermatitis and erythema multiforme, with no specific diagnostic biomarkers, rendering it prone to misdiagnosis. Furthermore, skin reactions in elderly bedridden patients are easily overlooked. The multidisciplinary team first confirmed the patient’s long-term medication history of parecoxib injection, followed by the identification of typical skin lesions, including extensive generalized blisters, epidermal detachment, and mucosal involvement. After excluding differential diagnoses such as allergic dermatitis and bullous dermatosis, the diagnosis of TEN was confirmed in accordance with the area of epidermal detachment relative to the BSA. The patient presented with advanced age, epidermal detachment involving over 30% of the BSA, and was complicated by hypoalbuminemia, electrolyte disorders, and long-term bedridden status, with a SCORTEN score of 3, indicating a high risk of mortality and adverse complications.

### 2.3 Treatment and clinical outcomes

**Figure 1:**
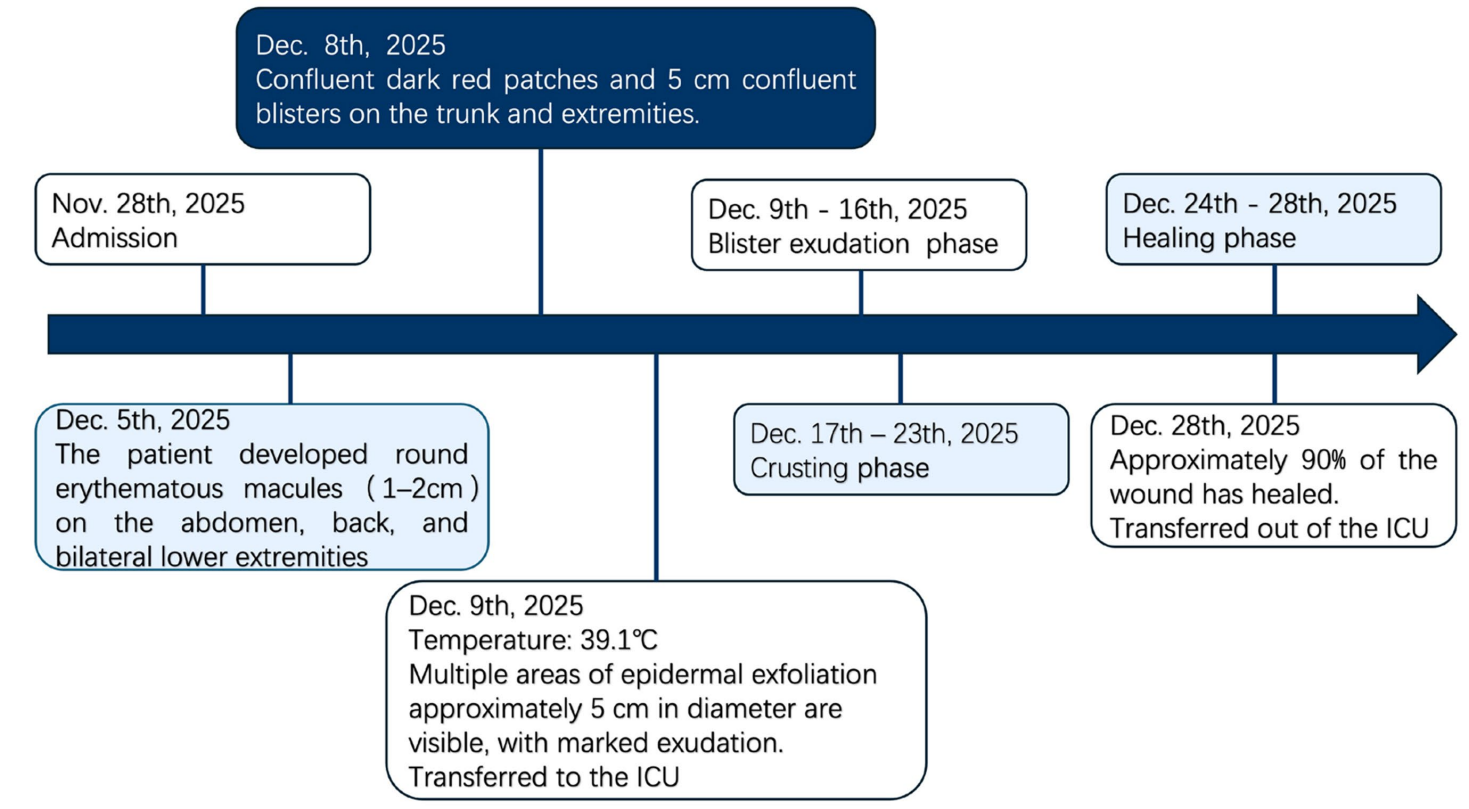
Disease course.

A multidisciplinary team consisting of the Department of Critical Care Medicine, Department of Orthopedics, Department of Dermatology, Department of Infectious Diseases, and Department of Clinical Nutrition was established to formulate the treatment regimen. Immediately after admission to the ICU, all suspected sensitizing medications, including parecoxib injection and ceftazidime for injection, were discontinued. In accordance with the DDG S3 Guideline (8), intravenous human immunoglobulin (IVIG) combined with corticosteroids was administered for anti-inflammatory therapy, along with albumin and plasma support, to rapidly control systemic inflammation and correct hypoalbuminemia and electrolyte disorders. Immunomodulatory and Anti-Inflammatory Therapy: An initial loading dose of 5 g IVIG was administered, followed by an adjusted dose of 20 g (qd) for 5 consecutive days, with an initial infusion rate of 0.4 g/kg/h. Methylprednisolone 40 mg was given by intravenous bolus twice daily (bid), which was adjusted to 80 mg (qd) for pulse therapy on December 15. Symptomatic and Supportive Care: Hypokalemia was corrected with 2 g of potassium chloride sustained-release tablets orally (tid), combined with slow infusion of 10 mL of 15% potassium chloride diluted in 40 mL of 0.9% sodium chloride solution. 10 g of human serum albumin was administered intravenously (qd), coupled with fresh frozen plasma infusion, to correct hypoalbuminemia and elevate plasma colloid osmotic pressure. After 17 days of treatment and nursing care in the ICU, upon ward transfer on December 28, partial generalized cutaneous erythema of the patient had turned dark yellow, with no new blisters or exudation observed. 1 cm × 1 cm skin islands emerged in multiple wound beds, which gradually expanded and coalesced, accompanied by a significant reduction in infection markers. The disease course is illustrated in Figure 1.

### 2.4 Follow-up and clinical outcomes

The patient’s BSA of skin involvement decreased from 55 to 5%, with a wound healing rate of approximately 95%. No new blisters, exudation, or secondary infection occurred. The mucosa of the oral cavity, lips, and eyes healed well, with no severe ocular complications such as eyelid synechiae or visual impairment. The patient’s body temperature returned to normal, inflammatory markers decreased significantly, electrolyte and albumin levels were essentially normalized, and vital signs remained stable. The patient’s cutaneous pain and pruritus were significantly relieved. She was able to cooperate with mild postural mobilization, with complete resolution of intraoral discomfort and feeding-related distress and maintained good treatment compliance. After transfer to the rehabilitation ward, continuous monitoring of wound healing was performed. No disease recurrence or exacerbation was observed, and inflammatory markers, liver and renal function, and electrolyte levels all remained within the normal reference range. During the entire treatment course, the patient did not develop severe adverse drug reactions. No adverse events including catheter-related infection, hypostatic pneumonia, pressure injuries, or severe ocular complications occurred. No secondary wound injury, accidental eschar avulsion, or other related incidents were documented.

## 3 Nursing care

According to the SCORTEN scoring system (9), the patient had a total score of 3 points (1 point for age 84 years >40 years; 1 point for 55% BSA epidermal detachment >10% BSA; 1 point for BUN 15.3 mmol/L > 10 mmol/L), with a corresponding expected mortality rate of 30–50%. The patient had generalized epidermal detachment with copious exudation, extensive mucosal involvement and complete skin barrier disruption, which, compounded by advanced age, fracture-related bedridden status and other comorbidities, conferred an extremely high risk of wound infection and poor wound healing. For this high-risk TEN patient, a meticulous, individualized nursing regimen centered on wound care was adopted, with multidimensional interventions implemented to enhance the prevention and management of complications (10).

### 3.1 Stage-specific nursing measures for skin wounds

**Figure 2:**
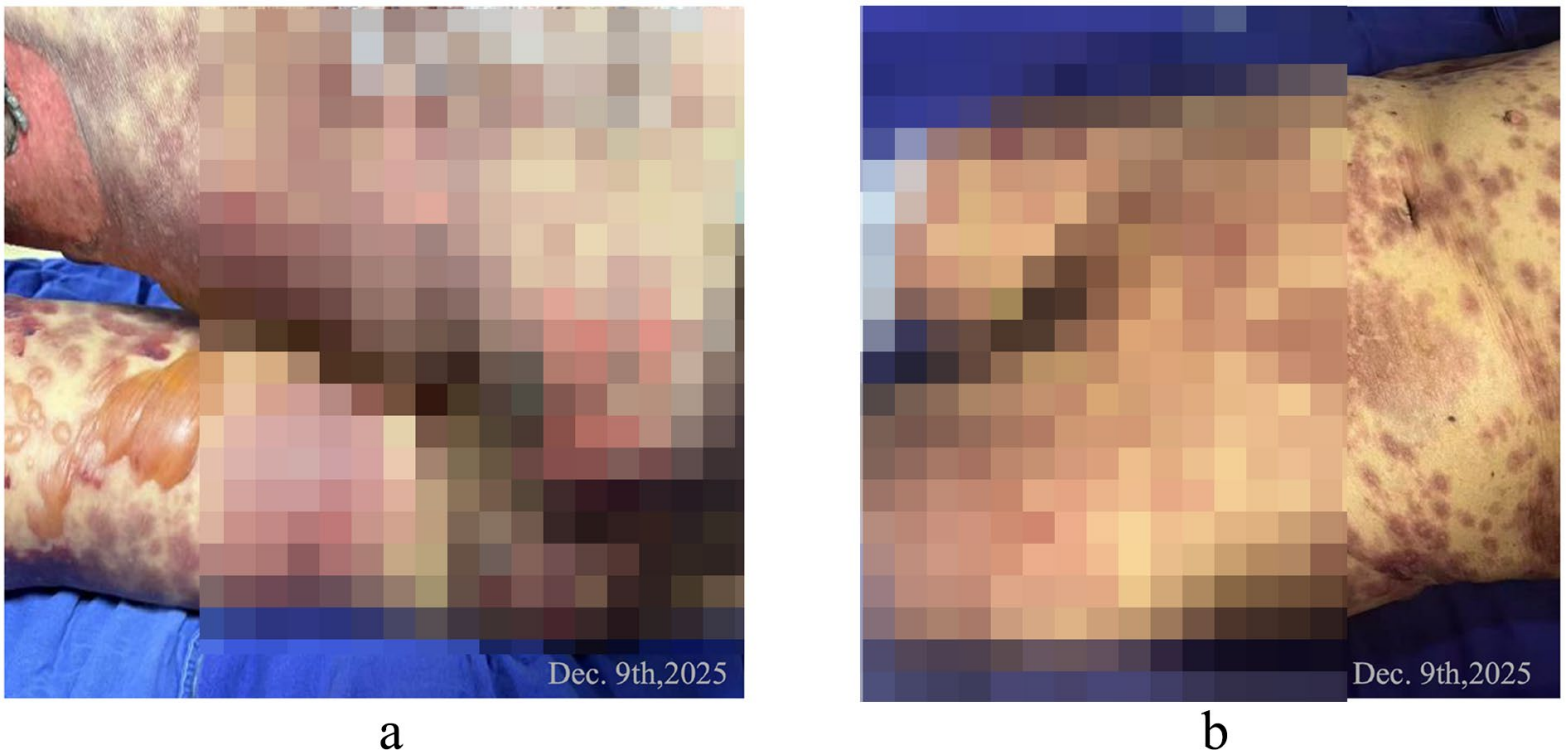
**(a)** Confluent blisters about 5 cm from both lower limbs, rupture of blisters on butts, and epidermal avulsion. **(b)** Large areas of redness and blisters on the abdomen, perineum, and lower limbs.

**Figure 3:**
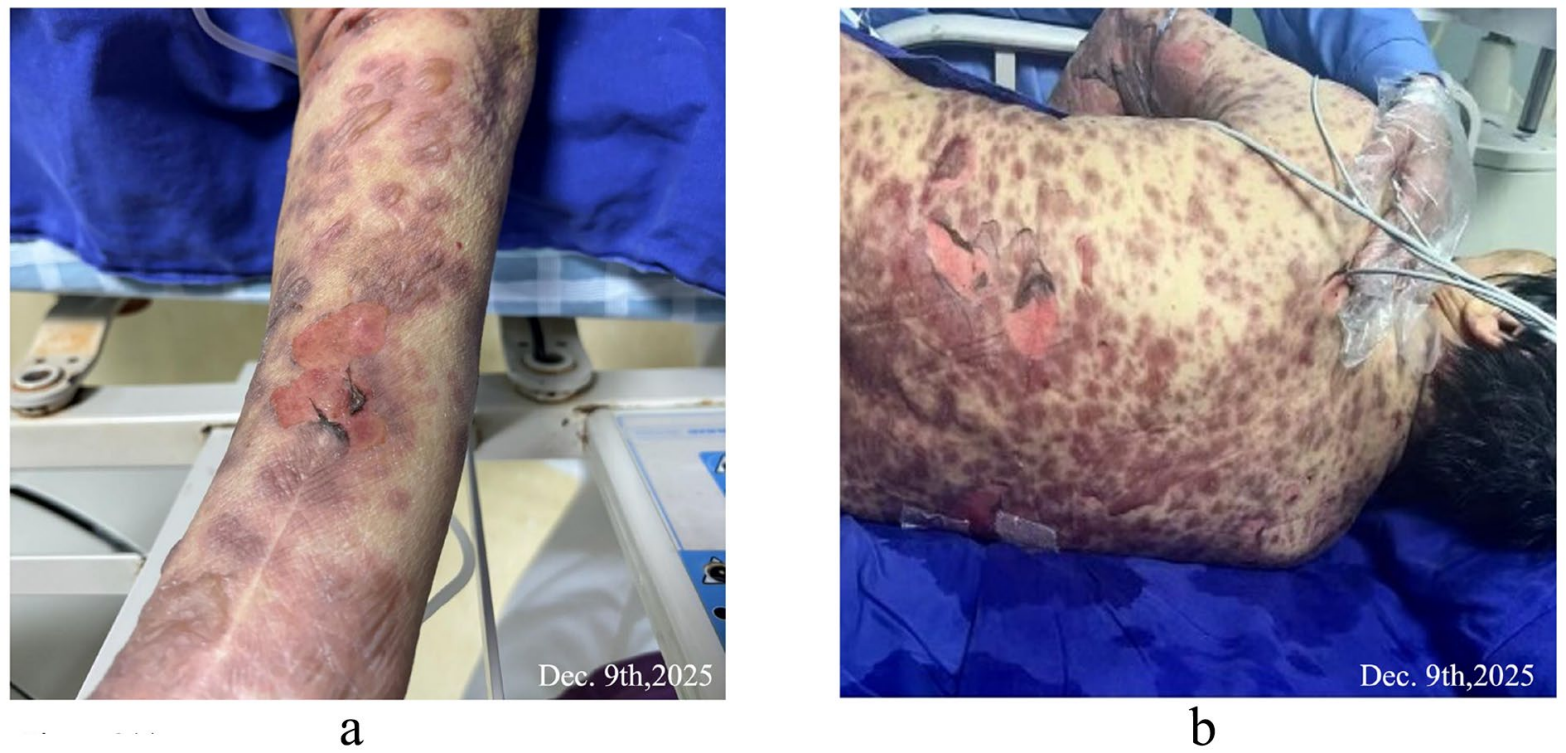
**(a)** Erythema and blisters on the upper limbs. **(b)** Back blisters were ruptured, epidermis avulsed, and large confluent erythema.

**Figure 4:**
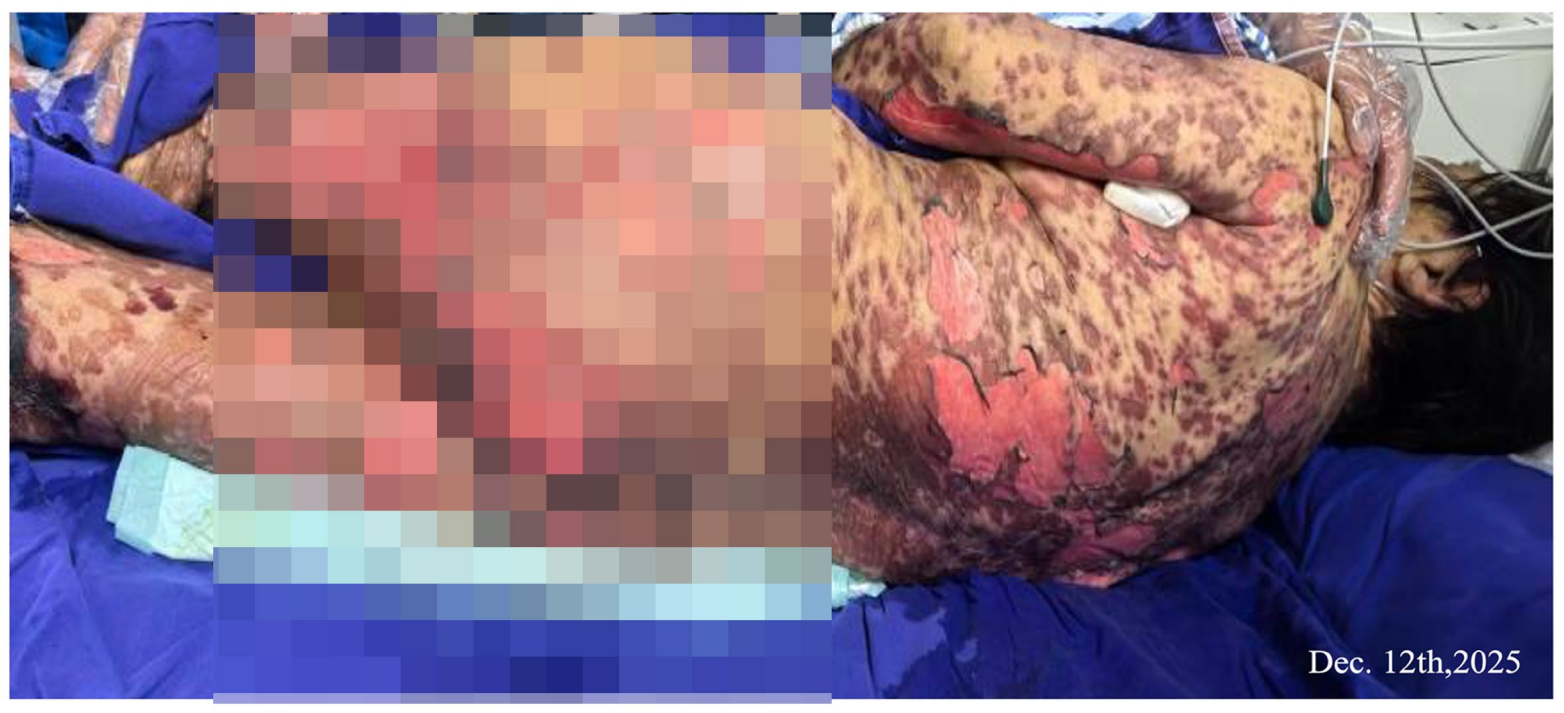
The skin exfoliation area was about 55%, with large areas of blisters bursting and exudation, and epidermal avulsion.

**Figure 5:**
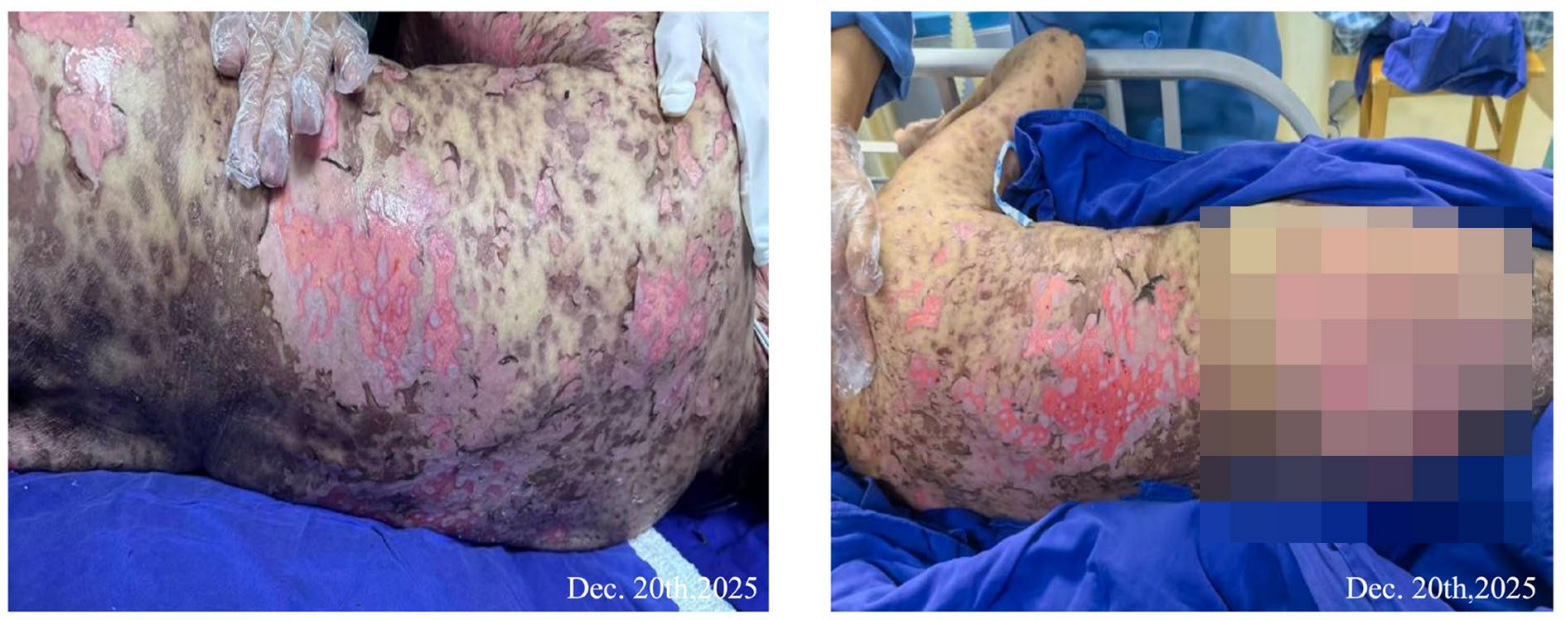
The area of skin exfoliation was reduced to 20%.

**Figure 6:**
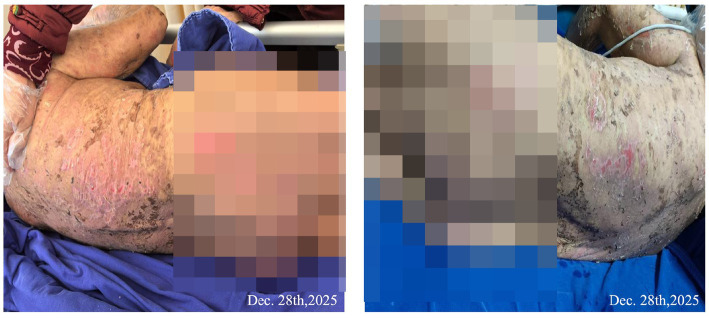
The skin exfoliation area of the back and caudal sacral ulcer was reduced to 5%.

In accordance with the natural healing process of skin wounds, we implemented targeted nursing interventions across three sequential phases (Blister-exudative phase, Crusting phase, and Healing phase), with strengthened nursing assessment and basic protection to guarantee the scientific rigor and continuity of care. The detailed staged wound care measures are shown in Table 2, Figures 2–6.

**Table 2:** Stage-specific nursing measures for skin wounds.

Nursing stage	Specific measures
Blister-exudation phase (Figures 2–4)	*1. Wound Assessment* The distribution, size, volume and color of blisters were accurately documented daily using the Lund-Browder chart, and the risk of infection was assessed in combination with pain scores, body temperature and wound surface temperature (11, 12). *2. Wound Cleansing and Disinfection* The patient’s body was wrapped with double-layered sterile therapeutic towels to absorb exudate, which were changed 3 times daily. Wound beds were gently irrigated with normal saline to remove necrotic epidermis and secretions. For large high-tension blisters with a diameter >1 cm, low-position puncture was performed to aspirate exudate, while the epidermal roof was preserved to cover the wound bed (13, 25). *3. Medication Administration* 200 IU of epidermal growth factor (EGF) solution was topically applied to the wound beds. 30 min after application, the wounds were covered with petrolatum gauze with excess ointment removed (25), twice daily. Wound secretions were collected synchronously for microbial culture at each dressing change. *4. Physical Intervention* Yellow light irradiation was administered once daily, with a wavelength of (590 ± 10) nm, power density of 20–40 mW/cm^2^, energy density of 20–40 J/cm^2^, a single treatment duration of 20 min, and a consistent irradiation distance of 30–50 cm (16)_._ The ward temperature and humidity were maintained at 28–32 °C and 50–60% respectively (12) to reduce transepidermal water loss from the wounds and enhance therapeutic efficacy. *5. Postural Management* An alternating pressure air mattress was used for pressure redistribution. The log-rolling technique (axial turning) was adopted for position changes, with sterile pillows used to support the extremities. Sterile therapeutic towels were used to assist with turning maneuvers simultaneously, to avoid cross-infection and secondary skin injury.
Crusting phase (Figure 5)	*1. Wound Management* The wound beds were kept dry, and moisturizing dressings were discontinued. The color, firmness, and presence of fluctuance of the eschar were monitored daily. In the event of subeschar pus accumulation, redness, swelling, and pain, the medical team was promptly notified for timely subeschar debridement. *2. Eschar Care* The patient and her family members were instructed to avoid eschar avulsion, keep fingernails trimmed and smoothed, and have the patient wear sterile gloves when necessary. *3. Skin Protection* The patient was wrapped with a single layer of sterile therapeutic towel. Gentle manipulation was strictly performed during towel changes to avoid friction against the eschar. *4. Pruritus Intervention* A mild moisturizing cream was gently applied to the intact skin surrounding the eschar to prevent scratching. Oral antipruritic medication was administered as prescribed by the clinician when necessary.
Healing phase (Figure 6)	*1. Healing Assessment* The extent of erythema regression, the rate of eschar shedding, as well as the color and elasticity of the newly regenerated epidermis were documented in detail, and the recovery of cutaneous sensation was assessed simultaneously. *2. Cleansing Care* The skin was gently cleansed with warm water, and the use of irritant soaps or shower gels was avoided. After cleansing, the skin was gently patted dry with sterile towels. *3. Ward Transfer Handover* Skin care records and imaging data were collated. A detailed handover of key nursing points, skin-sensitive areas and relevant precautions was conducted with the medical and nursing staff of the Department of Rehabilitation Medicine, to ensure the continuity of nursing care.

### 3.2 Mucosal and special site skin nursing

#### 3.2.1 Oral, labial and ocular nursing

**Figure 7:**
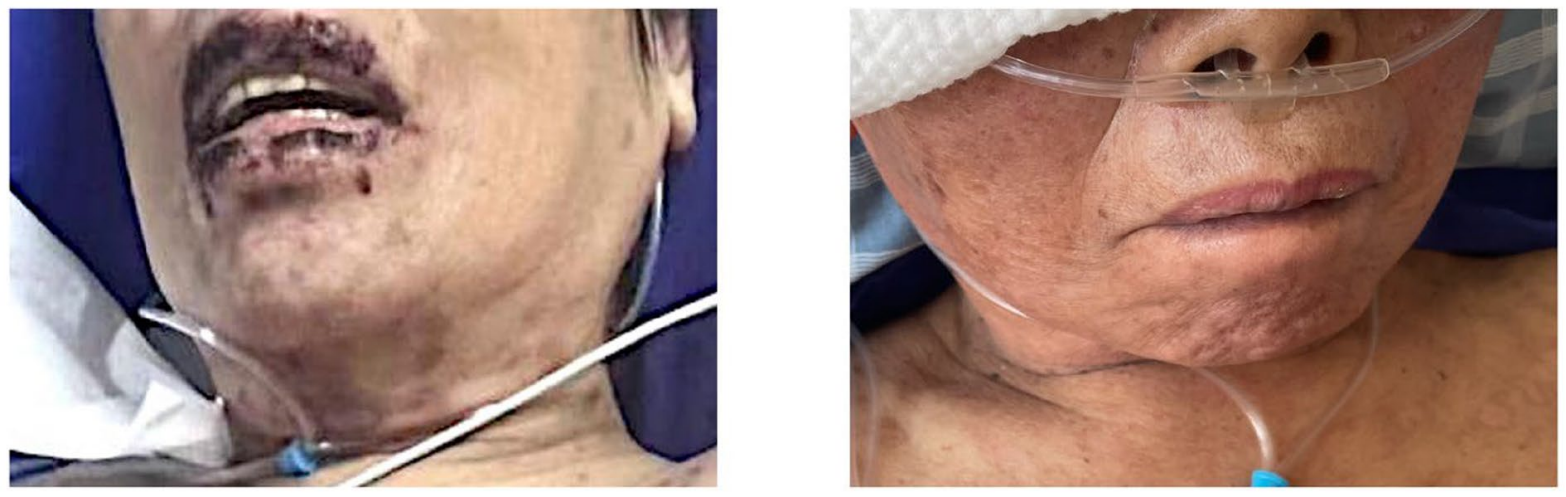
Before and after lip treatment.

The oral mucosa was gently wiped with cotton swabs moistened with 0.12% chlorhexidine oral rinse solution 4 times daily (11). Oral secretions were cleared with a disposable oral care sponge daily (12). Mouth rinsing with sodium bicarbonate injection was performed before and after meals to maintain oral moisture and prevent secondary infection (12, 13). For hemorrhagic erosions and black eschars on the lips, occlusive therapy was implemented: after topical application of tobramycin ointment, the affected area was covered with plastic wrap for moisturization, twice daily with 2 h per session (10, 12). The pre‑ and post‑treatment comparison of lip care is shown in Figure 7. The condition of the eyelid skin and conjunctiva was assessed daily (11, 14). In the acute phase, in the event of eyelid edema and increased secretions, the periorbital area was cleansed with cotton swabs moistened with sterile normal saline, and tobramycin eye drops were administered as prescribed twice daily to prevent conjunctivitis and eyelid synechiae (15). In the remission phase, periorbital cleansing with clean water was performed once daily (14). Protective goggles were worn to protect the eyes and periorbital skin during yellow light irradiation treatment (16).

#### 3.2.2 Perianal and perineal nursing

Given the patient’s bedridden status and perianal skin involvement, an indwelling urinary catheter was placed to minimize urine contamination (10, 13). The urethral meatus was cleansed 3 times daily and kept dry. The urinary catheter and drainage bag were replaced weekly under strict aseptic technique to reduce the risk of catheter-associated urinary tract infection (CAUTI). A colostomy bag was applied to collect feces when the patient felt the urge to defecate, and the perianal skin was maintained clean and air-permeable for the rest of the time. After removal of the colostomy bag, the perianal skin was gently cleansed with warm water. Strict hand hygiene and aseptic technique were implemented before and after all procedures to avoid wound irritation caused by fecal exposure.

### 3.3 Medication nursing and venous access nursing

#### 3.3.1 Medication nursing

In accordance with the DDG S3 Guideline: Diagnosis and Treatment of Epidermal Necrolysis (Stevens–Johnson Syndrome and Toxic Epidermal Necrolysis) (8), human immunoglobulin combined with methylprednisolone was administered to suppress the systemic inflammatory response and halt the progression of epidermal necrosis. Strict verification of the drug name, dosage, expiration date, and the absence of turbidity or precipitation was performed before infusion, and continuous cardiac monitoring was implemented to track vital signs throughout the infusion process. ① Low Molecular Weight Heparin (LMWH): Injections were administered exclusively to uninvolved skin, with daily rotation of injection sites to avoid subcutaneous ecchymosis caused by repeated injections at the same site. No compression was required after injection, and rubbing was strictly prohibited to prevent hematoma secondary to drug extravasation. ② Fresh Frozen Plasma (FFP) and Albumin: Strict verification of blood type, cross-matching results and plasma characteristics was completed before infusion. Infusion was initiated within 30 min, with the rate adjusted from slow to fast, and each infusion lasting no less than 30 min. The infusion line was flushed with normal saline after completion of infusion. Serum albumin levels and coagulation function were monitored to evaluate the efficacy of supplementation. ③ Intravenous Potassium Supplementation: 10 mL of 15% potassium chloride diluted in 40 mL of 0.9% sodium chloride solution was administered via a midline catheter, maintained at 10–15 mL/h with a syringe pump. Direct bolus injection was strictly prohibited. Serum potassium levels were monitored daily to maintain them within the normal reference range of 3.5–5.5 mmol/L.

#### 3.3.2 Intravenous access care

**Figure 8:**
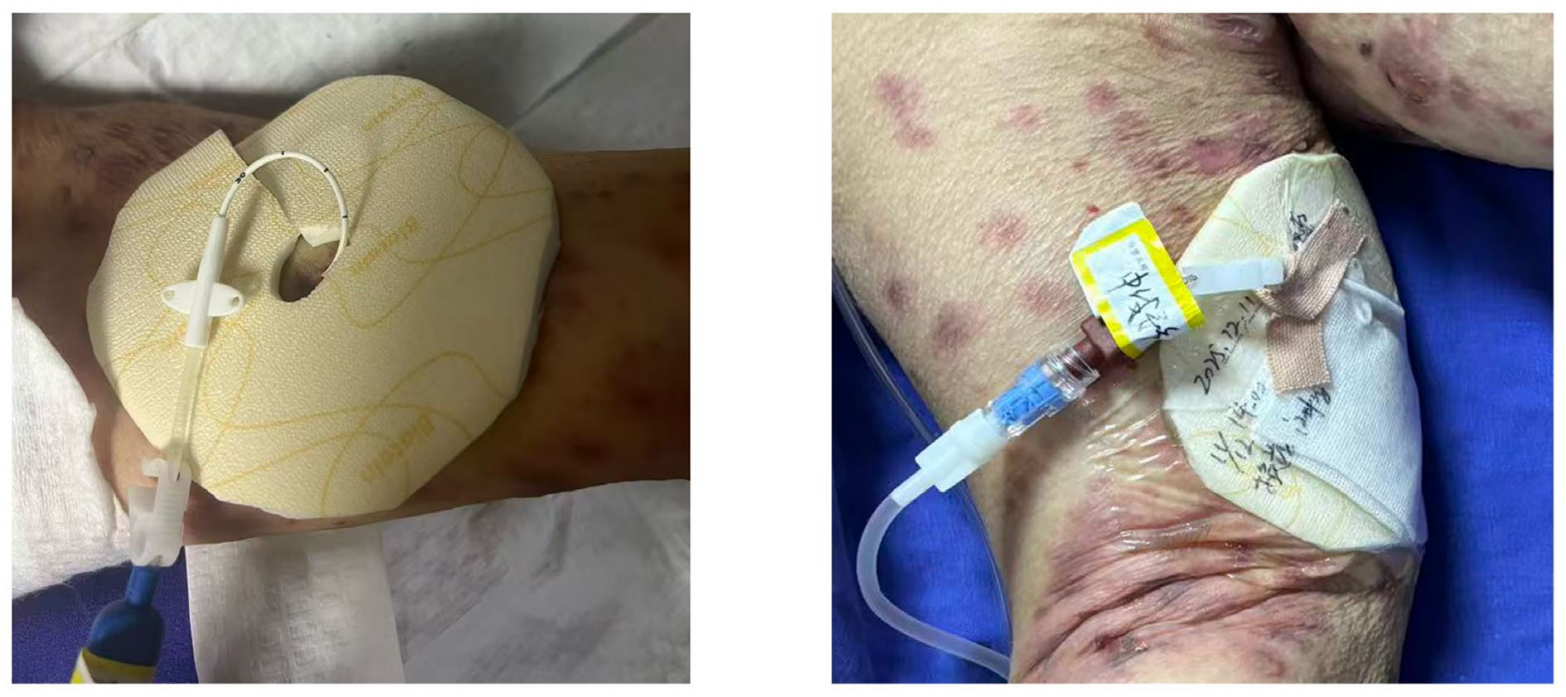
Beyertan non-adhesive foam dressing + 3M transparent application fixation scheme.

Given the patient’s advanced age and poor vascular access conditions, as well as the clinical requirement for infusion of immunoglobulin, antibiotics and blood products, a midline catheter was indwelled in the area with minimal skin lesions on the right upper extremity after assessment by an Intravenous (IV) Therapy Specialist Nurse (17). The catheter had an indwelling length of 17 cm and an external length of 5 cm, with the tip positioned in the axillary vein. A fixation regimen combining Biatain adhesive-free foam dressing and 3M transparent film dressing was adopted as follows: Epidermal growth factor was applied around the puncture site for skin protection; the foam dressing was trimmed to 8 cm × 8 cm, with a 1 cm × 1 cm circular hole cut in the center to accommodate the catheter, which facilitated visualization of the skin surrounding the puncture site and oozing at the puncture site; the foam dressing was placed under the catheter and covered with a 10 cm × 11.5 cm 3M transparent film dressing to secure the catheter with the minimal adhesive area. The catheter was labeled with the catheterization date, indwelling length and operator information. The puncture site was assessed daily, and the dressing was replaced promptly in case of loosening, contamination or moistness. The tension-free application technique was strictly implemented during all dressing changes (see Figure 8).

### 3.4 Infection prevention and control

The patient was placed in a single-room laminar flow sterile isolation ward, with a continuously operating air purification system for air decontamination, and the air bacterial colony count in the ward was monitored daily. Strict protective isolation measures were implemented: only 1 designated family member was allowed a 30-min visit per day. All visitors were required to change into sterile gowns, caps, masks and gloves, comply with hand hygiene protocols, and were strictly prohibited from contacting the patient’s wounds and medical supplies. The floor, bed units and other environmental surfaces were wiped with chlorine-containing disinfectant twice daily. All items in contact with the patient were either single-use disposable products or for the exclusive use of the patient, with prompt disinfection performed after use. Medical waste and domestic waste were strictly sorted and stored separately. During all invasive procedures, strict aseptic technique and hand hygiene protocols were followed. Sterile gloves and masks were worn throughout the entire operation, and disposable sterile instruments were used (17).

## 4 Discussion

### 4.1 Disease onset

According to the SCORTEN scoring system (9), age over 40 years and epidermal detachment involving more than 10% of the BSA are independent risk factors for disease deterioration and mortality in patients with TEN. The patient in this case was 84 years old, with a skin involvement area of up to 55% BSA. Her bedridden status due to fracture resulted in impaired local blood circulation and further damage to the skin barrier function. Advanced age led to impaired systemic immune function, decreased metabolic capacity, and poor drug tolerance, which contributed to the rapid progression of the disease. Meanwhile, the early clinical manifestations of TEN lack specificity and are easily confused with common skin diseases such as allergic dermatitis and erythema multiforme, which leads to delayed diagnosis and treatment and increases the difficulty of nursing management (3). After the multidisciplinary team established the definitive diagnosis of TEN, we reviewed the patient’s medication history and highly suspected that the disease was induced by the 23-day consecutive administration of parecoxib injection after the patient’s surgical operation. Therefore, for elderly patients with polypharmacy and concomitant trauma, in the event of unexplained cutaneous erythema and blisters with poor response to conventional treatment, the possibility of TEN should be highly suspected. Nursing staff should timely assist clinicians in completing relevant examinations to achieve early identification and early intervention.

### 4.2 Skin nursing

Cutaneous wound care is the cornerstone of nursing management for TEN, which directly determines the clinical prognosis of the disease. In this study, individualized nursing interventions were implemented based on the characteristic manifestations of different stages of wound healing. During the blister-exudative phase, low-position puncture was performed to aspirate blister exudate while preserving the epidermal roof, combined with epidermal growth factor solution and petrolatum gauze, which effectively reduced wound exudation and lowered the risk of infection. Yellow light irradiation was administered to accelerate wound healing. Turning maneuvers were assisted with sterile therapeutic towels. For one thing, this avoided cross-infection caused by direct contact between the operator’s hands and the patient’s skin, as well as secondary skin injury caused by pulling or dragging the patient’s skin during turning. For another, it evenly distributed the patient’s body weight across the sterile therapeutic towels, which not only reduced frictional force, but also alleviated the patient’s pain. During the crusting phase, the core focus was eschar protection to prevent scar hyperplasia caused by eschar avulsion. During the healing phase, emphasis was placed on skin barrier repair and standardized ward transfer handover to ensure the continuity of nursing care. Previous studies have confirmed that staged nursing interventions can significantly improve the quality of wound healing and reduce the incidence of infection in TEN patients (10). The scientific validity and clinical practicability of the nursing strategies adopted in this study have been verified through clinical practice.

### 4.3 Mucosal and special site skin nursing

The oral and labial mucosa are the most commonly affected sites in this disease (18). Oral care with 0.12% chlorhexidine combined with sodium bicarbonate can effectively inhibit the growth of oral flora and reduce the incidence of infection. For labial care, occlusive therapy was applied to create a closed moisturizing environment and promote the healing of labial mucosal erosions. Ocular complications have a high incidence in patients with TEN (19). In this study, prophylactic ocular nursing interventions were implemented, which effectively prevented severe complications such as visual impairment. The patient’s perineal mucosal condition was jointly assessed by ICU physicians, dermatologists and nursing staff (14, 17), and an indwelling urinary catheter was placed only after the integrity of the perineal mucosa was confirmed. The indwelling urinary catheter effectively isolated urine exposure and facilitated the nursing staff’s monitoring of the patient’s urine volume and characteristics. Fecal collection with a colostomy bag not only effectively blocked fecal irritation to the perianal skin, but also avoided mechanical injury induced by repeated insertion and withdrawal of the traditional bedpan, thus reducing the risk of wound infection and urinary tract infection (11).

### 4.4 Selection and maintenance of venous access

An Intravenous (IV) Therapy Specialist Nurse conducted a comprehensive assessment of the patient’s vascular access conditions and disease characteristics and opted for midline catheter placement in the right upper extremity, rather than a Central Venous Catheter (CVC) or Peripherally Inserted Central Catheter (PICC). Compared with CVC, the midline catheter has a superficial puncture trajectory and is technically easier to perform. It can effectively avoid severe complications such as pneumothorax and vascular injury, and the catheterization site does not restrict the patient’s mobility while reducing the risk of catheter contamination (20). Compared with PICC, the midline catheter has a smaller diameter, shorter length, and soft material, which causes less irritation to the vascular intima and skin, with a higher puncture success rate (20). Meanwhile, the midline catheter has a routine indwelling duration of 2–4 weeks (21), which is well matched with the patient’s treatment cycle. Taken together, compared with CVC and PICC, the midline catheter was the optimal venous access option for this patient. A fixation regimen combining Biatain adhesive-free foam dressing and 3M transparent film dressing was adopted, which effectively protected the impaired skin, absorbed exudate (22), and avoided direct adhesion between the transparent film dressing and the skin. Compared with the traditional single 3M film dressing, this regimen prevents skin traction that may lead to secondary skin injury and reduces the incidence of iatrogenic Medical Adhesive-Related Skin Injury (MARSI). In addition, enhanced routine catheter maintenance and strict implementation of aseptic technique were performed to ensure a safe and patent venous access, which underpinned the safe and uninterrupted delivery of the patient’s treatment.

### 4.5 Nursing experience

Recent case reports on TEN nursing have indicated that multidisciplinary collaboration, staged wound care, and infection prevention and control are consensus-recommended nursing strategies. However, few studies have developed individualized nursing regimens tailored to elderly patients, with comprehensive coverage of skin care, mucosal care, venous access management and other key aspects. Combined with this case and domestic and international research evidence (23, 24), the key nursing insights for elderly patients with TEN are summarized as follows: ① For elderly patients often have multiple underlying comorbidities and poor treatment tolerance. Nursing care should take into account both the primary disease and underlying comorbidities and implement individualized nursing regimens. ② For skin care should strictly follow the staged principle, focus on the protection of impaired skin and the avoidance of secondary injury, and combine physical therapy and pharmacotherapy to improve wound healing outcomes. ③ For venous access selection, comprehensive assessment of vascular access conditions and treatment requirements should be conducted. Priority should be given to access routes with minimal irritation to the skin and blood vessels, and innovative dressing fixation regimens should be adopted to protect the skin. ④ Nursing staff should routinely assess the patient’s medication history, family history and allergy history. During the administration of high-alert medications, regular assessment of the patient’s skin and mucous membranes should be performed to facilitate early identification and differential diagnosis. ⑤ For infection prevention and control, full-process protective isolation should be implemented, and aseptic technique should be strictly enforced throughout all nursing procedures. ⑥ Great importance should be attached to the psychological care of patients, while attention should also be paid to the psychological status of the nursing team, so as to enhance the humanistic connotation and professional quality of nursing services.

## 5 Reflection and prospect

This case is a rare case of severe toxic epidermal necrolysis (TEN) in an 84-year-old elderly patient with a SCORTEN score of 3 and multiple underlying comorbidities. After 17 days of ICU treatment, the patient achieved a 95% wound healing rate, which was significantly superior to the reported clinical outcomes of similar elderly patients, verifying the scientific validity and clinical effectiveness of the nursing strategy proposed in this study. This study also identified the unique challenges in the nursing management of elderly patients with TEN. Advanced age leads to impaired immune function and delayed wound healing in patients, while underlying comorbidities and extensive skin damage further increase the difficulty of nursing procedures and venous access maintenance. The evidence-based nursing interventions adopted in this study addressed the above challenges from multiple dimensions. However, this study still has limitations. For instance, no systematic geriatric psychological assessment tool was introduced. In subsequent studies, psychometrically validated geriatric psychological assessment tools with confirmed reliability and validity, such as the 15-item Geriatric Depression Scale (GDS-15) and the Hospital Anxiety and Depression Scale (HADS), can be introduced to systematically assess and address the psychological needs of patients, and to develop more targeted psychological intervention programs. In addition, this study lacks long-term prognostic follow-up of the patient, and the sample size of the single-case analysis is limited. Therefore, the effectiveness of the innovative nursing measures needs to be further verified. In the future, it is necessary to construct a multidisciplinary standardized nursing pathway for elderly patients with severe TEN, introduce precise assessment tools to improve the nursing system, and conduct large-sample multicenter studies to strengthen the evidence-based basis. Meanwhile, a full-cycle rehabilitation nursing system should be established, specialized training for the nursing team should be strengthened, and nursing strategies should be continuously optimized, so as to improve the nursing quality and short- and long-term prognosis of elderly patients with TEN.

## Data Availability

The original contributions presented in the study are included in the article/supplementary material, further inquiries can be directed to the corresponding author.
